# An Amyloid Core Sequence in the Major Candida albicans Adhesin Als1p Mediates Cell-Cell Adhesion

**DOI:** 10.1128/mBio.01766-19

**Published:** 2019-10-08

**Authors:** Vida Ho, Philippe Herman-Bausier, Christopher Shaw, Karen A. Conrad, Melissa C. Garcia-Sherman, Jeremy Draghi, Yves F. Dufrene, Peter N. Lipke, Jason M. Rauceo

**Affiliations:** a Department of Sciences, John Jay College of the City University of New York, New York, New York, USA; b Institute of Life Sciences, Université Catholique de Louvain, Louvain-la-Neuve, Belgium; c Biology Department, Brooklyn College of the City University of New York, Brooklyn, New York, USA; University of Texas Health Science Center

**Keywords:** functional amyloid, adhesion, cell wall, nanodomain, β-aggregation, adhesion, beta-aggregation

## Abstract

The human fungal commensal Candida albicans can become a serious opportunistic pathogen in immunocompromised hosts. The C. albicans cell adhesion protein Als1p is a highly expressed member of a large family of paralogous adhesins. Als1p can mediate binding to epithelial and endothelial cells, is upregulated in infections, and is important for biofilm formation. Als1p includes an amyloid-forming sequence at amino acids 325 to 331, identical to the sequence in the paralogs Als5p and Als3p. Therefore, we mutated Val326 to test whether this sequence is important for activity. Wild-type Als1p (Als1p^WT^) and Als1p with the V326N mutation (Als1p^V326N^) were expressed at similar levels in a Saccharomyces cerevisiae surface display model. Als1p^V326N^ cells adhered to bovine serum albumin (BSA)-coated beads similarly to Als1p^WT^ cells. However, cells displaying Als1p^V326N^ showed visibly smaller aggregates and did not fluoresce in the presence of the amyloid-binding dye Thioflavin-T. A new analysis tool for single-molecule force spectroscopy-derived surface mapping showed that statistically significant force-dependent Als1p clustering occurred in Als1p^WT^ cells but was absent in Als1p^V326N^ cells. In single-cell force spectroscopy experiments, strong cell-cell adhesion was dependent on an intact amyloid core sequence on both interacting cells. Thus, the major adhesin Als1p interacts through amyloid-like β-aggregation to cluster adhesin molecules in *cis* on the cell surface as well as in *trans* to form cell-cell bonds.

## Abstract (precis)

Microbial cell surface adhesins control essential processes such as adhesion, colonization, and biofilm formation. In the opportunistic fungal pathogen Candida albicans, the agglutinin-like sequence (*ALS*) gene family encodes eight cell surface glycoproteins that mediate adherence to biotic and abiotic surfaces and cell-cell aggregation. Als proteins are critical for commensalism and virulence. Their activities include attachment and invasion of endothelial and epithelial cells, morphogenesis, and formation of biofilms on host tissue and indwelling medical catheters. At the molecular level, Als5p-mediated cell-cell aggregation is dependent on the formation of amyloid-like nanodomains between Als5p-expressing cells. A single-site mutation to valine 326 abolishes cellular aggregation and amyloid formation. Our results show that the binding characteristics of Als1p follow a mechanistic model similar to Als5p, despite its differential expression and biological roles.

## INTRODUCTION

Fungal cell wall adhesins govern attachment to host surfaces and are essential for colonization of host tissue (1). Candida albicans is the most common human fungal pathogen and resides in the gastrointestinal and genitourinary tracts. Common cases of candidiasis include genital and oral infections. In some cases, candidiasis causes mortality and morbidity in immunocompromised individuals (2, 3). The mechanisms underlying adhesin function are relevant to understanding C. albicans pathogenesis, because colonization and invasion begin with adherence to host surfaces.

The agglutinin-like sequence (*ALS*) family includes eight genes, each encoding a cell wall-bound adhesin (4, 5). Als proteins mediate adhesion to host surfaces and may share binding targets between family members. *ALS1* was the first C. albicans adhesin gene discovered, and when expressed in a Saccharomyces cerevisiae surface display model, it mediates formation of large aggregates and flocs, as well as binding to endothelial cells (6, 7). Als1p plays a major role in C. albicans adhesion, including binding to human epithelial and endothelial cells and abiotic surfaces such as silicone and plastic (6, 8, 9). Also, normal biofilm and hyphal development require Als1p (10, 11). It is also key to interactions with bacteria and other yeasts in mixed biofilms (8, 12–15). Furthermore, C. albicans *als1Δ/Δ* homozygous mutants show decreased virulence, and *ALS1* expression is often used as a surrogate marker for virulence (11, 16, 17). Thus, Als1p function is a key surface determinant for C. albicans pathogenesis.

Hoyer and Hecht have proposed that the *ALS5* locus arose as a fusion of *ALS1* and *ALS6* (18). Als1p and Als5p have N-terminal immunoglobulin (Ig)-like invasin domains that are 70% identical, and they have overlapping but not identical sequence specificities for peptide ligands (8, 19–22). The T domains of wild-type Als1p (Als1p^WT^) and Als5p^WT^ have identical 108-amino-acid sequences, and each contains an ^325^IVIVATT β-aggregation core sequence (21, 23). C terminal to the T domain is a series of 36-residue tandem repeats, with the number of repeats varying between paralogs and between allelic versions of each paralog (24). The tandem repeats mediate hydrophobic effect binding to diverse ligands, including Als proteins themselves (i.e., homotypic binding [13, 25, 26]). With 20 tandem repeats in this allele of Als1p (6) versus only 6 repeats in Als5p (23), there is potentially greater hydrophobic surface exposed in each Als1p molecule. The C-terminal glycosylated stalks of Als1p and Als5p are different in length and in sequence. A C-terminal glycosylphosphatidylinositol (GPI) addition signal is cleaved in the endoplasmic reticulum (ER) as a GPI anchor is added. The GPI-bound form is excreted to the exterior face of the plasma membrane, where the GPI glycan is cleaved, and the remnant is covalently linked to cell wall glucan (5). Therefore, the mature forms of Als adhesins are anchored to the cell wall and have active domains for peptide binding, amyloid formation, and hydrophobic effect interactions.

When Als5p is expressed in an S. cerevisiae display model, amyloid formation greatly potentiates cell-cell aggregation (27, 28). A short amyloid-forming sequence from human Aβ protein can also potentiate activity when substituted into Als5p (29). Inhibition of amyloid formation with amyloid-perturbing compounds or peptides severely attenuates cell-cell aggregation and biofilm formation (27, 28, 30). These effects are also seen in C. albicans cells treated to maximize expression of Als1p (28). Als5p-mediated aggregation is reduced in cells expressing a single-site substitution mutant Als5p^V326N^ (28). This substitution preserves the conformation and binding activities of Als5p but severely attenuates amyloid formation, cell-to-cell binding, macroscopic cellular aggregation, and biofilm formation (27, 28, 31, 32). The sequence identity in the T region of Als1p (hereafter designated Als1p^WT^) and Als5p predicts that the properties might be similar, and both adhesins show high amyloid-forming potential in the homologous sequence at residues 325 to 331 (IVIVATT in both proteins), suggesting that activity of Als1p^WT^ is also amyloid dependent. Therefore, we constructed the homologous V326N mutation in Als1p and tested its phenotype using fluorescence, quantitative cell aggregation, and atomic force microscopy (AFM) assays (33, 34).

## RESULTS

### Cell surface localization of Als1p^WT^ and Als1p^V326N^.

**FIG 1:**
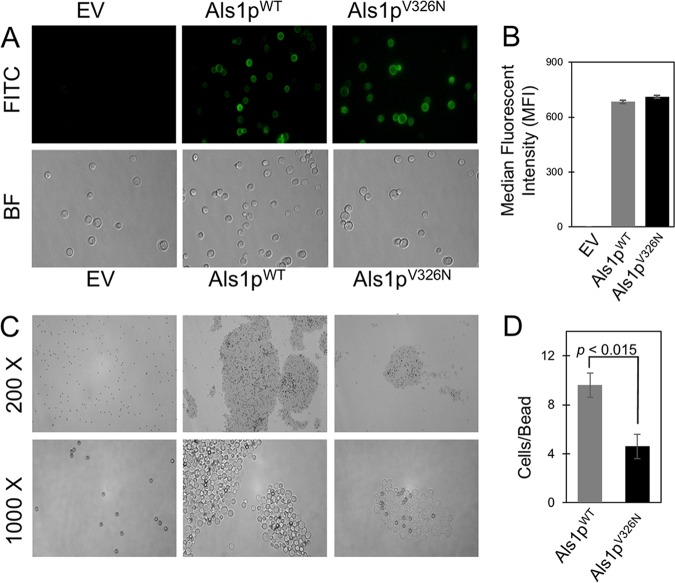
Cell surface localization and activity of Als1p^WT^ and Als1p^V326N^. (A) Intact cells were treated with anti-V5-FITC antibodies. Bright-field (BF) and fluorescent (FITC) photographs were taken of S. cerevisiae cells containing the empty vector (EV), expressing Als1p^WT^, and expressing Als1p^V326N^. All cells were viewed at a total magnification of ×1,000. (B) Quantitative analyses of Als1p^WT^ and Als1p^V326N^ expression levels. Als1p^WT^ and Als1p^V326N^ cells treated with anti-V5-FITC antibodies were quantified by flow cytometry, and mean fluorescence was determined. All samples were prepared in triplicate for statistical analysis. (C) Effect of V326N mutation on adherence to denatured BSA. S. cerevisiae cells with an empty vector or expressing Als1p^WT^ or Als1p^V326N^ were incubated with denatured BSA-coated magnetic beads (dark spheres, 1-μm diameter) and visualized under bright-field microscopy. Pictures were taken of cells observed at ×200 magnification (top row) and ×1,000 magnification (bottom row). (D) Quantitative analysis of cell-to-bead ratios. The values are means ± standard deviations (error bars) from three independent experiments.

An immunofluorescence assay was conducted to determine whether the Als1p protein reached the cell surface. Intact, unfixed cells expressing either Als1p^WT^ or Als1p^V326N^ were incubated with fluorescein isothiocyanate (FITC)-conjugated anti-V5 antibodies (anti-V5-FITC antibodies), washed, and viewed by fluorescent light microscopy. Both cell types fluoresced, demonstrating that the V5 epitope tag did not inhibit protein folding and localization (Fig. 1A). Flow cytometry showed no significant difference in the mean fluorescence of cells expressing the two forms of Als1p (Fig. 1B). Because the fluorescence of Als1p^WT^ and Als1p^V326N^ was similar, the mutation did not affect Als1p surface expression levels.

### Aggregation analysis of Als1p^WT^ and Als1p^V326N^.

We performed aggregation assays with bovine serum albumin (BSA)-coated beads to determine the effect of the V326N mutation on Als1p function (Fig. 1C). Cellular aggregation of Als1p^WT^ and Als1p^V326N^ was visually different. For cells expressing Als1p^V326N^, the aggregates were smaller but more numerous than for cells expressing Als1p^WT^. Specifically, there was no difference in the number of cells bound directly to beads, but there was less cell-to-cell binding, so the cell-to-bead ratio was lower (Fig. 1D). Thus, our findings show that a V326N mutation had a significant impact in cellular aggregation assays.

### Cell surface amyloid formation.

**FIG 2:**
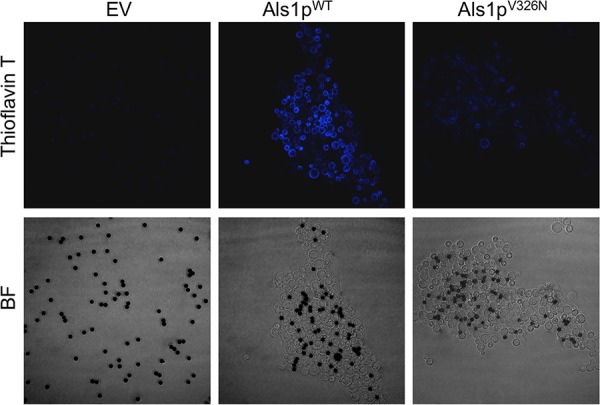
Effect of V326N mutation on amyloid formation. Cell surface amyloid formation was monitored with ThT in adhesion assays with BSA-coated magnetic beads (dark spheres, 1-μm diameter). Confocal microscopy was used to examine S. cerevisiae cells containing the empty vector (EV), cells expressing Als1p^WT^, and cells expressing Als1p^V326N^. Pictures were taken at 1,000× total magnification. Overall, the aggregates were of similar size to those shown in Fig. 1C. For the purposes of fluorescence comparison, we illustrate aggregates of Als1p^WT^ and Als1p^V326N^ that are of similar size.

Aggregation and other processes that expose cells to extension force activate formation of amyloid nanodomains on the surfaces of S. cerevisiae cells expressing Als5p or C. albicans cells (27, 28, 35, 36). We tested for the presence of surface amyloid nanodomains in S. cerevisiae cells expressing Als1p^WT^ or Als1p^V326N^. We used Thioflavin-T (ThT), which is a fluorescent indicator of the presence of amyloid. Cells were aggregated in the presence of ThT (100 nM) and then imaged. Cells expressing Als1p^WT^ were brighter than cells expressing the Als1p^V326N^ form of the protein, and there was no fluorescence of cells harboring an empty vector (Fig. 2).

### Detection of single Als1 proteins on cell surfaces.

**FIG 3:**
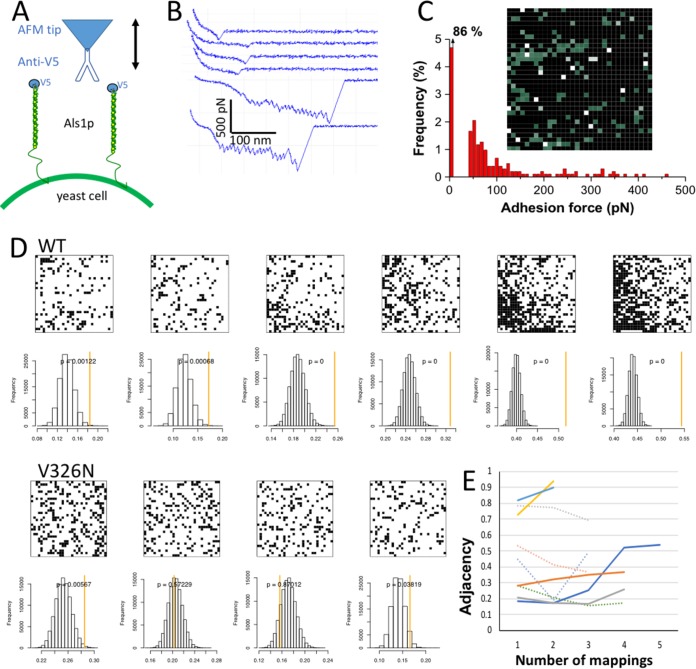
Single-molecule force spectroscopy on cells expressing Als1p. (A) Cartoon showing AFM configuration for single-molecule force spectroscopy (SMFS) with a molecule of anti-V5 bound to the tip and V5-labeled Als1p displayed on the surface of a live yeast cell. The tip was used to probe an array of 1,032 pixels within a 1 × 1 μm area on the cell surface. (B) Force-distance curves from such a mapping. The four top curves represent the majority of positive mappings with weak interactions that rupture at an extension force of 100 pN or less. The two bottom curves show strong interactions characterized by multiple force peaks corresponding to sequential unfolding, from left to right of the T domain, tandem repeats, and the Ig-invasin domains in Als1p (31, 39). (C) Rupture force histogram and map of Als1p occurrence in the probed area. The map shows green pixels wherever the probe bound to the cell, and white pixels for those events with rupture forces of ≥250 pN. (D) Maps and adjacency analysis for successive mappings of one area of a cell expressing Als1p^WT^ (top) and Als1p^V326N^ (bottom). The histograms show the distributions of adjacencies from 10^5^ simulations each at the same pixel density as the map above. The measured adjacency for each map is shown by the orange line. (E) Measured adjacencies on serial mappings for cells expressing Als1p^WT^ (solid lines) or Als1p^V326N^ (dotted lines).

Using spatially resolved single-molecule force spectroscopy (SMFS [37]), we probed single adhesins on yeast cells. A V5 epitope tag at the N-terminal end of Als1 proteins enabled us to detect single adhesins using AFM tips terminated with anti-V5 antibodies (Fig. 3A). Figure 3B shows representative force curves recorded between the antibody-tip and the surfaces of Als1p^WT^ yeast cells. A moderate proportion (16%) of force curves showed adhesion signatures that we attribute to the detection of single Als proteins. In force maps, proteins tended to form very few clusters, yielding a minimum protein surface density of ∼140 proteins/μm^2^. Two different force signatures were observed, i.e., low-adhesion force curves (∼99%) displaying single small adhesion forces (85 ± 40 pN [mean and standard deviation {SD}] from a total of 395 curves on three cells) at fairly short rupture distances (10 to 150 nm), and high-adhesion force curves (<1%) showing sawtooth patterns with multiple large force peaks (331 ± 38 pN; three cells) and long ruptures (150 to 400 nm). A map of a 1-μm^2^ region of the cell surface is shown in Fig. 3C.

Remapping the same 1-μm^2^ area of the surface yielded an increase in the number of adhesins detected and an increase from 0.2% to 2.6% in the fraction of saw-toothed unfolding curves typical of unfolding of successive protein domains (37). The high-adhesion force curves showed sawtooth patterns with multiple large force peaks (698 ± 209 pN maximum force and 298 ± 93 nm rupture distance from a total of 64 curves on three cells). This allele encodes an Als1p protein with a length of 1,560 amino acids, and each amino acid contributes 0.36 nm to the contour length of a fully extended polypeptide chain. Our measured rupture lengths correspond to about 50% of the expected lengths for fully extended proteins.

### Cell surface adhesin clustering.

The ^325^IVIVATT^331^ sequence in Als5p mediates adhesin clustering on the cell surface in response to extension force applied in an AFM (28, 35, 38). We therefore determined whether the sequence shows similar activity in Als1p^WT^. We used a statistical approach to quantify the clustering by determining the frequency of Als1p^WT^ molecules in a pixel immediately adjacent to another Als1p-occupied pixel. We then compared the frequency of this adjacency to 10^5^ simulations of random maps, each at the same surface density of adhesins as the experimental map. The Als1p^WT^ was clustered initially at greater than random expectation, and the clustering increased with successive mappings (Fig. 3D, top). Therefore, there was statistically significant clustering induced by successive rounds of extension force applied in the AFM. We also mapped the clustering of the nonamyloid mutant Als1p^V326N^. There was no significant clustering of the Als1p^V326N^ adhesin on remapping (Fig. 3D, bottom). The mean adjacency values for Als1p^WT^ consistently increased with the number of mappings (Fig. 3E, solid lines), whereas the values decreased or varied randomly for Als1p^V326N^ cells (dotted lines). Also, the *P* values for clustering decreased to 0 for cells expressing Als1p^WT^, i.e., the clustering was greater than in any of the 10^5^ simulations (Fig. 3D, top, yellow lines; see also Fig. S2 in the supplemental material). In contrast, *P* values for Als1p^V326N^ cells were uniformly within the distributions of the randomized maps (Fig. 3D, bottom, yellow lines; Fig. S2). Therefore, Als1p^WT^ clustered on the cell surface at greater than random frequency, whereas Als1p^V326N^ clustered near the frequencies expected for random associations.

10.1128/mBio.01766-19.2 FIG S2 Clustering analyses for successive mappings (left to right) of V5-Als1p on the surfaces of live cells. On each map, black pixels denote regions where a V5 epitope was detected. Beneath the map is a graph showing the mean adjacency value for the map (orange line) and a histogram of adjacency values for 10^5^ simulations. The *P* value for the measured adjacency is also given for each graph. (A and B) Cells expressing Als1p^WT^ (A) or cells expressing Als1p^V326N^ (B). Download FIG S2, PDF file, 0.3 MB. Copyright © 2019 Ho et al. 2019 Ho et al. This content is distributed under the terms of the Creative Commons Attribution 4.0 International license.

The peptide SNGINIVATTRTV has the same sequence as amino acid residues 322 to 334 of V326N variants of the adhesins, and it inhibits aggregation and biofilm formation in cells expressing Als1p^WT^ or Als5p^WT^ (28). We tested the peptide for inhibition of cell surface clustering. A cell was mapped twice to induce clustering, then treated with peptide (200 μg/ml), and mapped again. The peptide decreased clustering to a level similar to that seen in the initial map (Fig. S1A).

10.1128/mBio.01766-19.1 FIG S1 Effect of the antiamyloid peptide SNGINIVATTRTV. (A) Als1p^WT^ was repeatedly mapped on the surface of a cell, generating the data in the top row. The addition of peptide (14 μM) led to decreased clustering (bottom row). (B) SCFM analysis of cell-cell binding for a pair of cells (top row). The same cell pair was treated with 14 μM peptide (bottom row). Most cell-cell binding was abolished in the presence of peptide. Download FIG S1, PDF file, 0.2 MB. Copyright © 2019 Ho et al. 2019 Ho et al. This content is distributed under the terms of the Creative Commons Attribution 4.0 International license.

### Als1-mediated cell-cell adhesion forces.

**FIG 4:**
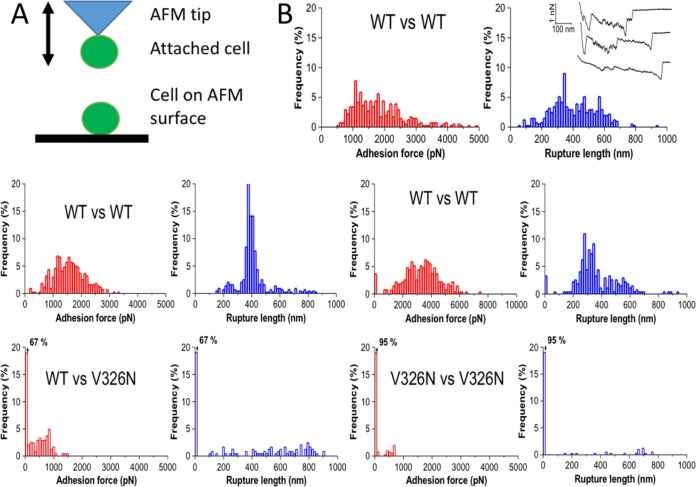
Force-distance analyses for cell pairs in SCFS. (A) Cartoon model of the AFM setup for these experiments. (B) Histograms of rupture forces and distances for five individual cell pairs. Each cell pair shows results of 500 adhesion-rupture trials. The Als1p version expressed by each cell type is labeled. The first cell pair has an inset showing three representative force-distance curves.

We used single-cell force spectroscopy (SCFS) to quantify the adhesion forces between single Als1p^WT^ cells. A single Als1p^WT^-expressing cell was attached to an AFM cantilever and then repeatedly brought into contact with another cell immobilized on the planchet surface in the AFM (Fig. 4A). In this configuration, adhesion between the two cells manifests as resistance to cell separation as the cantilever is withdrawn. Force-distance graphs showed “worm-like chain” characteristics (Fig. 4B, top right, inset) with successive sawtooth patterned peaks and recurring peak-to-peak distances of 9 nm, a value similar to Als5p (39). However, there were up to 40 peaks, corresponding to the unfolding of each of the 20 tandem repeat (TR) domains per interacting Als1p molecule (Fig. 4B). Adhesions between two cells expressing Als1p^WT^ showed many force-distance curves with large and complex adhesion signatures with a magnitude of 500 to 5,000 pN and a rupture length of 200 to 600 nm. The large rupture lengths suggest that cell-cell separation involves two (or more) interacting proteins between adhering cells. Cell-to-cell variations were observed which we attribute to differences in protein expression (Table 1).

**TABLE 1:** Characteristics of cell-cell adhesion in SCFS experiments

Cell 1	Cell 2	% of cell pairs binding	Mean rupture force (pN)^*a*^	Mean rupture length (nm)^*a*^	Mean maximum rupture force per pair (pN)	*N*^*b*^
Als1p^WT^	Als1p^WT^	97 ± 17	1,514 ± 1,166	507 ± 106	3,329	7
Als1p^WT^	Als1p^V326N^	33 ± 11	483 ± 104	393 ± 169	953	3
Als1p^V326N^	Als1p^V326N^	7 ± 4	517 ± 29	467 ± 29	667	3

a Values are means ± standard deviations for adhesions with nonzero force.

b Number of cell pairs assayed, with 500 trials per cell pair.

We also tested effects of the V326N mutation on cell-cell binding. Binding of a cell expressing Als1p^WT^ to a cell expressing Als1p^V326N^ led to threefold reductions in the probability of adhesion (from 97% to 33%), as well as mean rupture force (from 1,514 to 483 pN) in maximal rupture force (Table 1). When both cells expressed Als1p^V326N^, the adhesion probability was reduced to 7% without significant further change in rupture force or length (Table 1). In addition, most cell-cell adhesion was abolished in the presence of the antiamyloid peptide (Fig. S1B).

## DISCUSSION

The major C. albicans adhesin Als1p mediates multiple interactions and leads to multiple consequences for biofilm formation and pathogenesis. Among its known activities, its ability to aggregate C. albicans cells and to coaggregate with bacteria and other fungi are key for pathogenesis and infection (13, 40). In addition to binding diverse peptide ligands and l-fucose (22, 41), nonspecific binding activity of Als1p is dependent on the presence of tandem repeats, which mediate hydrophobic effect interactions with a variety of targets (25, 26). As we show here, the ability to form amyloid-like β-aggregates is key for formation of strong and specific homotypic interactions. A consequence would be persistent biofilms that resist antifungals and other palliative treatments.

Als1p mediates β-aggregation-dependent cellular aggregation as it does in its close paralog, Als5p (28, 30, 38). In each case, the sequence ^325^IVIVATT is essential, as discussed below. However, the context is different, and thus, the dependence of aggregation on the β-aggregation potential differs. Specifically, a V326N mutation reduces β-aggregation potential by >90% in each case, and in Als5p, it severely attenuates activity in aggregation assays (28). In Als1p, this same mutation reduces aggregate size by about 50% (Fig. 1). This remaining activity may reflect greater aggregation due to hydrophobic effect interactions between the 20 tandem repeat domains in Als1p versus the 6 domains in Als5p.

In single-molecule AFM experiments, Als1p^WT^ aggregated on the cell surface, whereas Als1p^V326N^ did not (Fig. 3). A novel statistical tool quantifies the clustering, and it shows frequencies far higher than expected from random associations. This result was similar to observations with Als5p, in which amyloid-like β-aggregation is necessary for clustering through interactions of the identical sequence on many molecules of the protein (28, 35, 38, 42).

Similarly, in cells expressing Als1p as in cells expressing Als5p, cell-cell binding was driven by β-aggregation. In aggregation assays mediated by either adhesin, the cells become bright if stained with the amyloid dye Thioflavin T at submicromolar concentration (Fig. 2) (30). For cells expressing Als1p as well as cells expressing Als5p, cell-to-cell binding is dependent on V326: the V326N variant shows highly reduced binding probability and bond strength (Table 1) (42). In fact, in Als1p-expressing cells, the probability of adhesion dropped 14-fold from 97% between cells expressing Als1p^WT^ to 7% for cells expressing Als1p^V326N^, and the cell-cell bond strength was reduced threefold, despite similarity in the cell surface concentration of the two forms (Fig. 1B). In heterologous adhesion between a cell expressing Als1p^WT^ and a cell expressing Als1p^V326N^, the probability was intermediate but the cell-cell binding force was weak, with a value similar to that between two cells expressing Als1p^V326N^.

These data strongly support the idea that in cells expressing Als1p as well as in cells expressing Als5p, cell-cell interactions are dependent on β-aggregation of homologous amyloid core sequences around V326. The adhesin clusters (*cis* interactions) increase the probability of intercellular bonding (*trans* interactions). As a result, strong *trans* bonds form between cells expressing Als1p^WT^, but not between an Als1p^WT^-expressing cell and an Als1p^V326N^-expressing cell. The presence of a sequence-specific antiamyloid peptide reduced surface clustering and cell-cell bonding to levels like those for Als1p^V326N^ cells (see Fig. S1 in the supplemental material). These findings are consistent with the idea that amyloid-like β-aggregation both in *cis* on the surface of a cell and in *trans* between cells is the basis for strong cell-cell bonding in both adhesins (42).

This similar adhesion mechanism leads to stronger bonding between cells expressing Als1p than in cells expressing Als5p. The difference may be biologically important and may be a basis of their different biological roles. In C. albicans, Als1p is expressed in planktonic cells in lag phase and also in hyphae under some conditions (13, 43, 44). Als1p is highly expressed in mouse models of infection and in artificial biofilms (14, 45). In contrast, Als5p is moderately expressed, and its expression can skew human macrophages toward the tolerant M2 state or lead to commensal-like interactions in a Caenorhabditis elegans infection model (46, 47). These independent biological roles are reflected in differences in the activities of Als1p and Als5p *in vitro.* Thus, the β-aggregation-prone amyloid core sequence IVIVATT is the basis for formation of amyloid-like β-aggregate bonds formed in *trans* between cells expressing homologous adhesins. Other regions of the two adhesins must modulate activity *in vivo* to facilitate their different roles in host-pathogen interactions.

## MATERIALS AND METHODS

### Construction of plasmid vectors and yeast strains.

Plasmid pYF-5 (6) was used as the template for PCR amplification. To produce the *V5-ALS1* plasmid, primers containing 5′-flanking NotI (forward primer) and XhoI (reverse primer) restriction site sequences were used to amplify the entire *ALS1* coding region without the signal sequence. The amplicon was gel purified, ligated to the pCR-Blunt II-TOPO vector (Invitrogen) and transformed in Escherichia coli XL10-Gold cells (Agilent) to produce plasmid pVH1. Plasmid DNA was extracted using a Qiagen plasmid extraction kit according to the manufacturer’s protocol, and the insert was sequenced (GeneWiz, South Plainfield, NJ). Plasmids pVH1 and pJL1 (which contains the N-terminal invertase secretion signal and V5 epitope tag [28]) were digested with XhoI and NotI restriction enzymes. The *ALS1* insert and pJL1 vector backbone were gel purified and ligated with T4 ligase to form plasmid pVH3. This plasmid expresses Als1p^WT^ when grown on galactose.

To construct the *V5-ALS1* V326N mutant plasmid, the *ALS1* Ig and T regions were amplified by PCR, ligated to the pCR-Blunt II-TOPO vector, and transformed into E. coli cells. Plasmid DNA was purified, and nucleotides corresponding to Val326 were mutated to encode Asn using the site-directed mutagenesis kit (Agilent) following the manufacturer’s protocol. The mutagenized plasmid (pCSR1) was transformed into E. coli and purified, and the insert was verified by DNA sequencing. To swap the mutant amyloid sequence into the *ALS1* sequence, pCSR1 was digested with SacII and AleI to release the Ig-T^V326N^ fragment and gel purified. pVH1 was similarly digested with SacII and AleI, and the plasmid vector without the Ig-T regions was gel purified. The mutant insert fragment was ligated to the pVH1 vector fragment to create plasmid pVH4. Plasmids pVH4 and pJL1 were digested with XhoI and NotI, and the complete *ALS1* insert fragment containing the V326N mutation was ligated to the pJL1 vector backbone to produce plasmid pVH5. pVH5 was amplified in E. coli and purified, and the insert was sequenced. This plasmid expresses Als1p^V326N^ when grown on galactose.

Plasmids pVH3 and pVH5 were transformed into S. cerevisiae strain W303-1A (*MAT***a** *leu2 ura3 ade2 trp1*). *ALS1* expression was induced by growth in complete synthetic medium without tryptophan and with galactose as the carbon source (CSM-Trp/Gal).

### Immunofluorescence assays.

Cells were grown to stationary phase in CSM-Trp/Gal medium. The cells were washed three times with 1 ml of Tris-EDTA (TE) (10 mM Tris Cl, 20 mM EDTA [pH 7.0]) buffer, and approximately 1 × 10^8^ cells were resuspended with 100 μl of TE buffer. Next, 1 μl of fluorescein isothiocyanate-conjugated anti-V5 antibody (1 mg/ml anti-V5-FITC; NOVUS Biologicals) was added to the cell suspension and incubated for 40 min in the dark. The cells were washed with 1 ml of TE buffer three times, resuspended in 100 μl of TE buffer, and viewed with a light microscope. Fluorescent cells were quantified using the Attune NxT flow cytometer (Life Technologies) as previously described (48). We gated out all events with fluorescence below the maximum value for unstained cells to account for cellular autofluorescence. Ten thousand events were collected for each sample, and the median fluorescent intensity (MFI) was determined. All samples were analyzed in triplicate, and experiments were performed at least three independent times. Data were analyzed using Attune NxT software v2.2, and statistical differences were analyzed using paired *t* tests in GraphPad Prism v5.01.

### Adhesion assays.

Adhesion assays were performed as previously described (28, 49). Briefly, Als1p-expressing S. cerevisiae cells were grown to stationary phase in CSM-Trp/Gal medium. Approximately 1 × 10^8^ to 3 × 10^8^ cells were harvested and washed three times in TE buffer. The cells were mixed with magnetic beads (approximately 100:1 cell-to-bead ratio) coated with heat-denatured bovine serum albumin (BSA) in TE. For adhesion assays using Thioflavin T (ThT), washed cells were vortexed for 1 min in 1 ml of 100 nM ThT-TE solution prior to adding magnetic beads to facilitate amyloid formation. The cells and beads were shaken at room temperature (∼20 to 25°C) for 30 min, and the aggregates were magnetically separated and washed three times in TE buffer. About 10 to 15 μl of cells and beads were spotted onto a microscope slide and viewed with a Nikon Eclipse E600 confocal light microscope. Pictures were taken at 200× and 1,000× total magnification. Samples treated with ThT were viewed at 1,000× total magnification with a Nikon Eclipse 90i confocal microscope with 408-nm excitation and 450-nm emission filters. The remaining cell-bead suspension was resuspended in 0.1 M NaOH and vortex mixed to disrupt cell-cell and cell-bead interactions. The cells and beads were counted on a hemocytometer. All samples were analyzed in triplicate, and experiments were performed at least three independent times. Statistical differences between experimental and control groups were determined in paired *t* tests.

### Single-molecule force spectroscopy.

Single-molecule force spectroscopy measurements were performed at room temperature (20°C) in phosphate-buffered saline (PBS), using a Nanoscope VIII multimode atomic force microscope (AFM) (Bruker Corporation, Santa Barbara, CA) and oxide sharpened microfabricated Si_3_N_4_ cantilevers (Bruker Corporation, Santa Barbara, CA). Cells were immobilized by mechanical trapping into porous polycarbonate membranes (Millipore), with a pore size similar to the cell size. After filtering a concentrated cell suspension, the filter was gently rinsed with buffer, carefully cut (1 cm × 1 cm), and attached to a steel sample puck (35). The mounted sample was transferred into the AFM liquid cell while avoiding dewetting. The spring constants of the cantilevers were measured by using the thermal noise method.

AFM tips were functionalized with anti-V5 antibodies (Invitrogen) using polyethylene glycol (PEG)-benzaldehyde linkers as described by Ebner et al. (50). Briefly, cantilevers were washed with chloroform and ethanol, placed in an UV ozone cleaner for 30 min, immersed overnight in an ethanolamine solution (3.3 g of ethanolamine into 6 ml of dimethyl sulfoxide [DMSO]), washed three times with DMSO and two times with ethanol, and dried with N_2_. The ethanolamine-coated cantilevers were immersed for 2 h in a solution containing 1 mg Acetal-PEG-NHS (*N*-hydroxysuccinimide) dissolved in 0.5 ml chloroform with 10 μl triethylamine, washed with chloroform, and dried with N_2_. Cantilevers were covered with a 200-μl droplet of a PBS solution containing anti-V5 (0.2 mg/ml) to which 2 μl of a 1 M NaCNBH_3_ fresh solution was added. After 50 min, cantilevers were incubated with 5 μl of a 1 M ethanolamine solution in order to passivate unreacted aldehyde groups, and then the cantilevers were washed with and stored in PBS 10 min later.

### Cluster analysis.

To distinguish whether the positive measurements were uniformly dispersed or concentrated into clusters, we implemented a simple measure of local clustering and used randomization and empirical *P* value distributions to assess the statistical significance of that measure. For each lattice, we calculated a clustering score by examining each positive cell and counting which cells in its immediate neighborhood were also positive. Neighborhoods were generally defined as the eight cells closest to the focal cell but were reduced for focal cells at the edges or corners of the sample area. The mean fraction of positive neighbors for each positive cell was then used as our clustering statistic. To evaluate its significance, we repeatedly generated randomly permuted samples of the same lattice, effectively scrambling the location of each positive cell. Applying the same clustering statistic to each of many such scrambled lattices produced a null distribution unique to each data set; we could then approximate a one-tailed *t* test by asking what fraction of the null distribution had a higher clustering statistic than the true data set did. We labeled these fractions as empirical *P* values to reflect the fact that they originate by reshuffling the actual data, rather than from a defined null distribution. A total of 100,000 replicates were performed for each data set to ensure sufficient resolution in the resulting empirical *P* values.

### Single-cell force spectroscopy.

Cell probes were prepared using triangular shaped tipless cantilevers (microlevers) (catalog no. NP-O10; Bruker Corporation) coated with bioinspired polydopamine wet adhesives. Cantilevers were immersed for 1 h in a 10 mM Tris buffer solution (pH 8.5) containing 4 mg ml^−1^ dopamine hydrochloride (99%; Sigma) and dried with N_2_ flow. Single cells were then attached onto the polydopamine-coated cantilevers using a Bioscope Catalyst AFM (Bruker Corporation). Hydrophobic substrates were prepared by immersing gold-coated substrates overnight in solutions of 1 mM 1-dodecanethiol (Sigma-Aldrich) (98%), rinsing them with ethanol, and drying them under N_2_. To have cell aggregates on the hydrophobic surface, we deposited a drop of a cell suspension and allowed it to sediment for 10 to 15 min, and the cells were covered with 4 ml of PBS. Then, the cantilever was brought into contact with an isolated cell for 3 min, and the obtained cell probe was then transferred over a cell aggregate for cell-cell force measurements. The nominal spring constant of the cantilever was ∼0.06 N m^−1^ as determined by the thermal noise method. Single-cell force spectroscopy measurements were performed at room temperature (20°C) in PBS, using a Bioscope Catalyst combined with an inverted optical microscope (Zeiss Axio Observer Z1 equipped with a Hamamatsu camera C10600 [Oberkochen, Germany]).
