# Supplementary material for: An Amyloid Core Sequence in the Major Candida albicans Adhesin Als1p Mediates Cell-Cell Adhesion
Source: mBio. 2019 Oct 8;10(5):e01766-19. doi: 10.1128/mBio.01766-19 (PMC6786869; doi:10.1128/mBio.01766-19)

Supplemental Figure S2. Clustering analyses for successive mappings (left to right) of V5-Als1p on the surface of live cells. On each map, black pixels denote regions where a V5 epitope was detected. Beneath the map is a graph showing the mean adjacency value for the map (orange line) and a histogram of adjacency values for 10^5 simulations. The *p* value for the measured adjacency is also given for each graph. A) cells expressing Als1p^{WT} B) cells expressing Als1p^{V326N}.

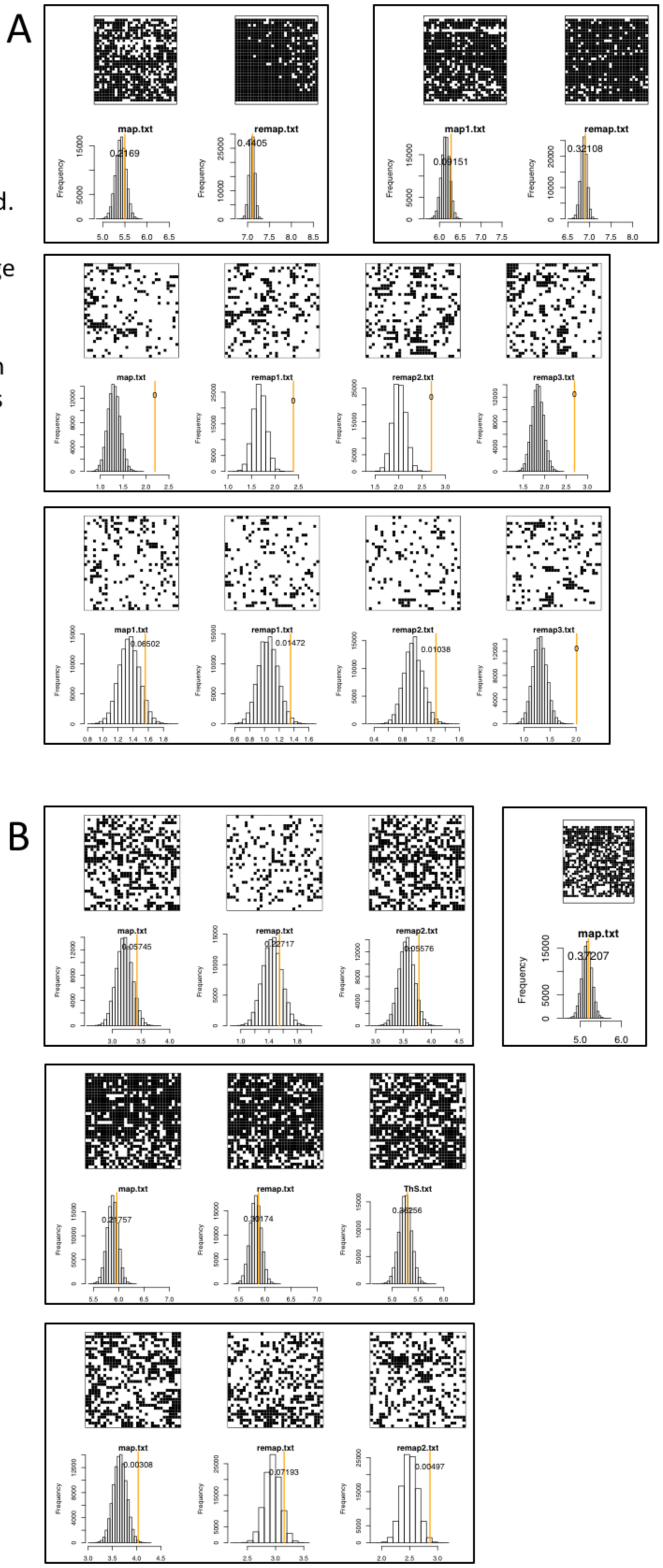

Supplement: FIG S2 [file mBio.01766-19-sf002.pdf]
