# Supplementary material for: An Amyloid Core Sequence in the Major Candida albicans Adhesin Als1p Mediates Cell-Cell Adhesion
Source: mBio. 2019 Oct 8;10(5):e01766-19. doi: 10.1128/mBio.01766-19 (PMC6786869; doi:10.1128/mBio.01766-19)
Supplement: FIG S1 [file mBio.01766-19-sf001.pdf]

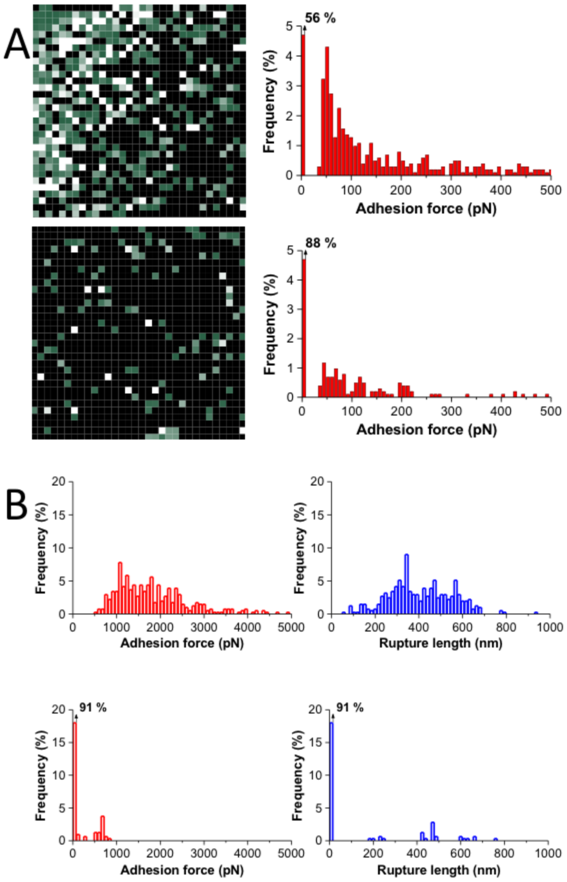

Supplemental Figure S1. Effect of the anti-amyloid peptide SNGINIVATTRTV. A) Als1p^{WT} was repeatedly mapped on the surface of a cell, generating the data in the top row. Addition of peptide (14 µM) led to decreased clustering (second row). B) SCFM analysis of cell-cell binding for a pair of cells (top row). The same cell pair was treated with 14 µM peptide (bottom row). Most cell-cell binding was abolished in the presence of peptide.
